# Upper limits on the extent of seafloor anoxia during the PETM from uranium isotopes

**DOI:** 10.1038/s41467-020-20486-5

**Published:** 2021-01-15

**Authors:** Matthew O. Clarkson, Timothy M. Lenton, Morten B. Andersen, Marie-Laure Bagard, Alexander J. Dickson, Derek Vance

**Affiliations:** 1 Department of Earth Sciences, ETHZ, 8092 Zurich, Switzerland; 2 Global Systems Institute, University of Exeter, Exeter, EX4 4QE UK; 3 School of Earth and Ocean Sciences, University of Cardiff, Cardiff, CF10 3AT UK; 4 School of Environment, Earth and Ecosystem Sciences, The Open University, Milton Keynes, MK7 6AA UK; 5 Department of Earth Science, University of Cambridge, Cambridge, CB2 3EQ UK; 6 Department of Earth Sciences, Royal Holloway University of London, Egham, TW20 0EX UK

**Keywords:** Carbon cycle, Element cycles, Palaeoceanography, Palaeoclimate, Marine chemistry

## Abstract

The Paleocene Eocene Thermal Maximum (PETM) represents a major carbon cycle and climate perturbation that was associated with ocean de-oxygenation, in a qualitatively similar manner to the more extensive Mesozoic Oceanic Anoxic Events. Although indicators of ocean de-oxygenation are common for the PETM, and linked to biotic turnover, the global extent and temporal progression of de-oxygenation is poorly constrained. Here we present carbonate associated uranium isotope data for the PETM. A lack of resolvable perturbation to the U-cycle during the event suggests a limited expansion of seafloor anoxia on a global scale. We use this result, in conjunction with a biogeochemical model, to set an upper limit on the extent of global seafloor de-oxygenation. The model suggests that the new U isotope data, whilst also being consistent with plausible carbon emission scenarios and observations of carbon cycle recovery, permit a maximum ~10-fold expansion of anoxia, covering <2% of seafloor area.

## Abstract (web summary)

The expansion of oceanic anoxia during the Paleocene Eocene Thermal Maximum has important implications for faunal turnover patterns and global biogeochemical cycles. Here the authors use uranium isotopes and a biogeochemical model to suggest that the areal expansion of anoxia must have been limited to 10-fold.

## Introduction

The Paleocene Eocene Thermal Maximum (PETM) is one of the best studied climate perturbation events in Earth’s history, but the driving mechanisms, environmental consequences, and recovery processes are still debated. The PETM is characterized by the input of isotopically light carbon, probably representing a mixture of mantle-derived carbon from large igneous province (LIP) activity and oxidation of organic carbon reservoirs^1–4^. These emissions resulted in warming of ~5 °C (ref. ^5^) and a negative carbon isotope (δ^13^C) excursion (CIE) of ~3–4‰, associated with rapid environmental deterioration including ocean acidification^6^, changes in hydrology and weathering regimes^7^, alteration of ocean circulation^8^, locally enhanced primary productivity^9,10^, and local de-oxygenation of the water column^7,11–15^.

There is increasing recognition that expanded oceanic anoxia is a defining consequence of past global warming events^16^, and that it is predicted to worsen under future climate projections^17^. Indicators of local de-oxygenation are common for the PETM, with the development of low-O_2_ conditions in open ocean locations and fully anoxic to euxinic (anoxic and sulfidic) environments in some continental shelf and restricted basin sites^7,11–15,18–20^. Water column de-oxygenation is often invoked as a contributor to benthic faunal turnover and extinction patterns^21,22^. Various mechanisms have been proposed to drive de-oxygenation during the PETM, including changes in deep ocean and basin ventilation, upwelling strength, temperature solubility effects, methane hydrate destabilization, and enhanced nutrient inputs from the continents^9,22–24^. Many of the characteristics and hypotheses for the PETM are shared with the Mesozoic Oceanic Anoxic Events (OAEs), allowing them to be compared for insights into the driving mechanisms of environmental catastrophe but also for understanding negative feedbacks that counter greenhouse gas forcing and enable climate recovery, such as silicate weathering and organic carbon burial^1,16,25^.

Despite extensive study, the overall global extent and severity of anoxia during the PETM is poorly quantified, making it difficult to meaningfully compare it to other periods of oceanic anoxia. This limitation is largely due to reliance on local redox proxies, which act as targets for spatially resolved Earth system models^15,20^, but yield patchy geographical information and conflicting interpretations^7^. Global-scale geochemical insights, from molybdenum (Mo) isotopes, suggest that euxinia was more prevalent during the PETM than the modern^11^ but less extensive than during Mesozoic OAEs^26^. Sulfur (S) isotope evidence has also been used to model a 10−20-fold increase in the volume of euxinic waters (with up to 0.5 mM H_2_S) during the PETM compared to the modern ocean^27^. Whilst these studies provide important insights into general redox characteristics, neither capture non-euxinic redox changes that are crucial for understanding faunal responses and biogeochemical feedbacks during climate perturbations.

To overcome these limitations, we present the first carbonate-associated uranium isotope data (^238^U/^235^U, reported as δ^238^U_CAU_; see “Methods”) for the PETM interval. Uranium solubility and isotope fractionation are strongly redox sensitive (commonly as U(VI) to U(IV)), with the preferential sequestration of ^238^U, relative to ^235^U, in reducing sediments^28,29^. Uranium reduction primarily occurs at or below the sediment–water interface, at a redox potential similar to that for iron reduction^30^. As such, authigenic U(IV) accumulation is a result of bottom-water and pore-water anoxia, but does not require euxinic conditions within the water column. With a long residence time in the modern ocean (~320–560 kyrs^31^), δ^238^U can trace global-scale changes in benthic anoxia^28^: events such as OAE 2 (ref. ^32^) and the Permo-Triassic Boundary^33,34^ are associated with clear shifts to lower δ^238^U_CAU_ values that are thought to reflect trends in seawater δ^238^U (δ^238^U_sw_) and can be quantitatively related to the expansion of anoxic sinks.

New δ^238^U_CAU_ and U/Ca data are presented for bulk carbonate leachates (see “Methods”) from three geographically dispersed pelagic localities that span the PETM: ODP Site 865 from Allison Guyot, equatorial Pacific Ocean (paleodepth ~1500 m), DSDP Site 401 on the continental slope of the Bay of Biscay (~2000 m), and ODP Site 690 from Maud Rise in the Southern Ocean (~2100 m)^35–37^. Samples are poorly lithified carbonate-rich sediments with a mixture of benthic and pelagic foraminifera, or carbonate nannofossil ooze, and show variable contributions from detrital clays^35–37^. The intermediate-depth equatorial Pacific is thought to have been mostly oxygenated before and during the PETM^14,20^, and δ^238^U_CAU_ is likely to reliably record δ^238^U_sw_. By contrast, the other two sites experienced varying degrees of de-oxygenation with observable effects on local U systematics^13,20^. The δ^238^U_CAU_ from Site 865 and published δ^13^C data^1^ provide constraints for an established carbon–phosphorus–uranium (C–P–U) biogeochemical box model^32^, based on the more comprehensive COPSE model^38^, to quantify the extent of seafloor anoxia during the PETM.

## Results

### Trends in U/Ca and U isotopes

**Fig. 1:**
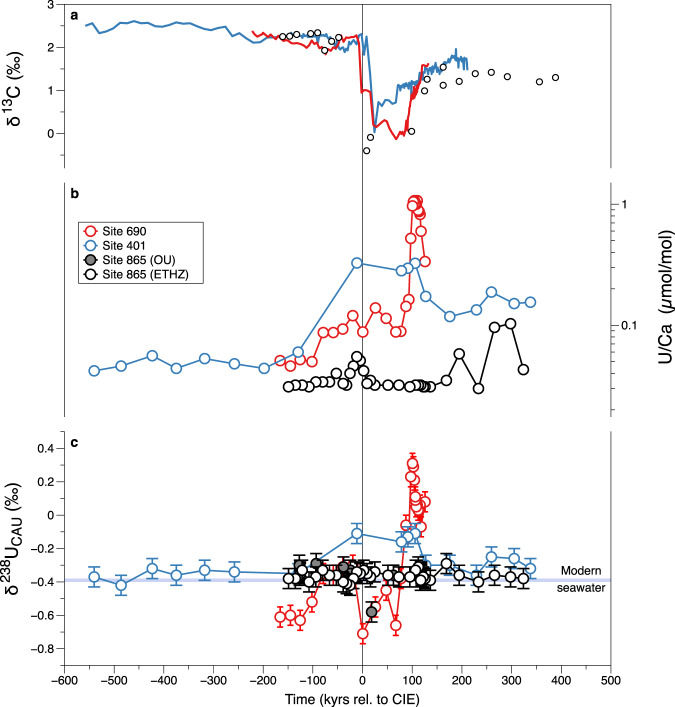
Uranium isotope and U/Ca data for pelagic PETM localities. **a** Bulk carbonate δ^13^C data from Site 401 (blue line; ref. ^1^) and Site 690 (red line; ref. ^4^), and *A. soldadoensis* δ^13^C for Site 865 (black circles; ref. ^53^). All data are plotted as a function of time relative to the onset of the carbon isotope excursion (CIE) using the ^3^He-based age model^68^ (see “Methods”). **b** U/Ca from carbonate leachates corresponding to δ^238^U_CAU_ plotted in **c**, with symbols colored by site as in **a**. Note a log scale is used in **b**. U/Ca are not shown for samples run at the Open University (OU; gray filled circles) due to incomplete carbonate digestion (see “Methods”). Error bars for δ^238^U_CAU_ (**c**) represent the external reproducibility of the NIST-SRM 1d limestone standard (±0.06‰, 2 SD, *n* = 14; see “Methods”). PETM δ^238^U_CAU_ data are compared to average modern δ^238^U_sw_ in **c** (−0.39 ± 0.01; blue shading)^28^.

All three sites exhibit stable background U/Ca that increases around the onset of the PETM (Fig. 1 and Supplementary Figs. 1–3). For Site 865, the increase defines a minor peak, from 0.02 to 0.05 µmol/mol, beginning ~20 kyrs before the PETM onset and extending to ~25 kyrs after (see “Methods” for age model details). Lower U/Ca values are seen for the remainder of the record, except for a few samples (up to ~0.1 µmol/mol) in the shallowest part of the core. For Site 401, U/Ca increases from 0.05 to 0.27 µmol/mol at the PETM onset and remains elevated for the next ~120 kyrs before decreasing again for the remainder of the record, but remaining above pre-PETM values. Larger U enrichments are seen at Site 690 where U/Ca increases from a background of ~0.05 µmol/mol to over 1 µmol/mol. At Site 690, the U enrichments occur in two main phases, firstly ~100 kyrs prior to the PETM onset, and, more significantly, later during the PETM recovery interval.

Sites 865 and 401 both have largely invariant δ^238^U_CAU_ values of −0.36 ± 0.06‰ (2 SD), before and after the PETM interval. Site 865 shows no resolvable variation in δ^238^U_CAU_ for the entirety of the record, except for a single anomalously low sample at −0.58‰, and there is no change in δ^238^U_CAU_ related to the minor peak in U/Ca (Fig. 1). Higher U/Ca at Sites 401 and 690 are positively correlated with δ^238^U_CAU_ (Fig. 1). Additionally, Site 690 shows lower pre-PETM values than the other sites and a return to these low values between intervals of higher U/Ca.

### The relationship of δ^238^U_CAU_ to seawater

Pre-PETM U/Ca are similar to modern bulk pelagic carbonates but higher than modern foraminifera^39,40^. These U/Ca match better with the higher partition coefficient of abiotic calcite compared to biogenic calcite^39^, suggesting that the majority of carbonate-associated U resides in syn-sedimentary authigenic cements^40^. Sites 865 and 401 demonstrate remarkable invariance and reproducibility in pre-PETM δ^238^U_CAU_, with an average of −0.36 ± 0.06‰ (2SD) that is similar to modern seawater (−0.39 ± 0.01‰; ref. ^28^). The simplest explanation for this uniformity is that these sites directly record contemporary δ^238^U_sw_, with little or no associated isotopic fractionation, as shown for some modern biogenic and abiotic calcites^41–43^. This implies that, under well-oxygenated conditions, U is incorporated into the carbonate lattice of syn-sedimentary cements as U(VI) whilst in communication with overlying seawater, and is not influenced by reducing conditions during early burial. The precise extent of U isotope fractionation into authigenic carbonate depends on the U(VI) speciation in the aqueous phase, which may lead to δ^238^U_CAU_ up to ~0.1‰ heavier than seawater^42^, so it is possible that Paleocene δ^238^U_sw_ could have been lower, down to ~ −0.46‰. Site 690 appears to also show invariant baseline values before the PETM, but with a lower δ^238^U_CAU_ of −0.64‰. This difference might reflect a higher proportion of adsorbed (isotopically light) U in the authigenic carbonate at Site 690, such as has been inferred for a modern pelagic bulk carbonate sample^40^, or U associated with planktic organic matter^44^ that could be incorporated into authigenic calcite. The same explanation could also apply to the single anomalously low value at Site 865.

### Diagenetic influences

The deep-sea core samples used for this study have experienced relatively simple diagenetic histories compared to carbonates used in the majority of paleo-studies using U isotopes, which to date have focused mainly on platform carbonates and/or outcrop material^32,33,45–47^. In the case of platform carbonates, that originally consisted of metastable aragonite or high-Mg calcite, diagenetic corrections are often applied to δ^238^U_CAU_ values^33,45–47^ to account for authigenic U(IV) uptake under reducing porewater conditions during early diagenesis (including recrystallization and cementation) that results in a high degree of scatter with a generally positively offset compared to seawater (~+0.27 ± 0.14‰; 1 SD)^48,49^. Previous work has suggested that some pelagic carbonates record lower δ^238^U_CAU_ signatures, closer to seawater values compared to platform counterparts, and thus do not require correction^32,41^. Similarly, biogenic calcite from brachiopod shells is thought to better retain primary δ^238^U_CAU_ signatures than metastable matrix components^43^. The better preservation of δ^238^U_sw_ in pelagic carbonate samples is likely due to the stable mineralogy of these sample types (low-Mg calcite), but also the generally low organic carbon accumulation rates and low fluid flow rates of pelagic settings^43^. Here, increases in δ^238^U_CAU_ and U/Ca at Sites 690 and 401, that likely reflect early diagenetic U(IV) uptake, are systematically tied to local environmental change related to the PETM hyperthermal (see below). Such patterns therefore reflect an integration of bottom-water and pore-water conditions, carrying information on local environmental deterioration, rather than a constant diagenetic process that requires correction. Given the correspondence of pre-PETM δ^238^U_CAU_ to near modern seawater values, we do not apply any diagenetic correction factor in order to reconstruct seawater compositions.

The main concern for the integrity of the presented datasets is for Site 865, where the planktic foraminifera are known to have undergone recrystallization, resulting in opaque calcite^50^, although the benthic species are well preserved^51^. Geochemical alteration of the planktic foraminifera resulted in a temperature-dependent offset in planktic foraminiferal δ^18^O, whilst δ^13^C of individual planktic specimens approach the bulk carbonate signature^52^ but still capture the structure of the PETM CIE^53^. By contrast, boron (B) isotopes (δ^11^B) appear to be well preserved^52,54^ but B/Ca is lower in recrystallized specimens, suggesting that although B is lost during recrystallization the remaining calcite-bound B did not exchange with diagenetic pore fluids^52^. Given the immobility of U in deeper (anoxic) burial environments, and the relatively closed nature of recrystallization at Site 865 (ref. ^52^), we would expect a similar preservation of primary U isotope signatures^43^. Regardless of any planktic foraminiferal recrystallization, it is important to recognize that the measurements presented here were performed on bulk carbonate leachates where the majority of U is thought to be present in syn-sedimentary cements. Thus, any recrystallization of the planktic foraminifera, that contribute only a fraction of the U mass balance, should have little impact on the bulk carbonate U isotope signatures. It is noteworthy that the minor U/Ca increase at Site 865 corresponds consistently with benthic foraminifera turnover patterns that are suggestive of de-oxygenation^51^ (see below; Supplementary Fig. 1), implying that early authigenic carbonate signatures are preserved during burial. Moreover, the replication of pre-PETM δ^238^U_CAU_ at Site 401 strongly suggests that the Site 865 record is not compromised by recrystallization.

### Influence of local de-oxygenation

**Fig. 2:**
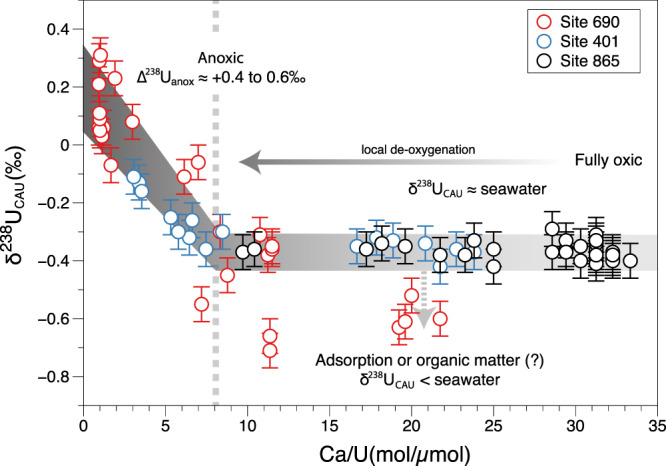
Relationship of δ^238^U_CAU_ and U concentrations. Note that U concentrations are expressed as Ca/U (inverted relative to Fig. 1) in order to emphasize the samples showing U enrichment (lower Ca/U) coupled to high δ^238^U_CAU_ at Sites 690 (red circles) and 401 (blue circles). These samples demonstrate a near-linear relationship when Ca/U < 8 mol/µmol, which are consistent with a Δ^238^U_anox_ of up to ~+0.6‰ relative to inferred δ^238^U_sw_. Data from Site 865 (black circles) and pre-PETM data from Site 401 that plot along the flat array of Ca/U likely record δ^238^U_sw_. δ^238^U_CAU_ values below inferred δ^238^U_sw_ at Site 690 could be caused by the adsorption of isotopically light U directly to calcite or repartitioned during organic matter re-mineralization. Error bars for δ^238^U_CAU_ represent the external reproducibility of the NIST-SRM 1d limestone standard (±0.06‰, 2 SD).

Close to the onset of the PETM, the three sites show very different responses in U systematics. Both Sites 690 and 401 record higher U/Ca correlated with higher δ^238^U_CAU_ (Fig. 1). In pelagic sediments, elevated U/Ca in foraminifera is thought to semi-quantitatively track overall authigenic U enrichment^55^ which is also likely to be reflected in the bulk carbonate data presented here. Together with the higher δ^238^U_CAU_ values, these trends indicate more reducing conditions and are consistent with other proxy evidence in these sediments, including benthic species turnover patterns^21,56^, barium accumulation rates (BARs)^10^, cerium anomaly data, and chromium isotopes^20^ (Supplementary Figs. 2 and 3; Supplementary Note 1). By contrast, Site 865 records only a minor increase in U/Ca, that is also coincident with local benthic faunal turnover evidence for increased food supply or lower O_2_ conditions^51^ (Supplementary Fig. 1; Supplementary Note 1), but with no resolvable change in δ^238^U_CAU_ (Fig. 1). These two behaviors can be clearly separated by examining the relationship between U concentrations and δ^238^U_CAU_ for the three localities, a relationship that is likely a function of localized redox conditions (Fig. 2). Note that in Fig. 2, U concentrations are expressed as Ca/U, as opposed to U/Ca in Fig. 1, to better identify the relationship of U enrichments coupled to high δ^238^U_CAU_. All the data for Site 865 and the pre-PETM data for Site 401 are characterized by substantial variability in U concentrations but none in δ^238^U_CAU_. By contrast, the PETM data from Site 401 and PETM-recovery data from Site 690 exhibit larger U enrichments (lower Ca/U) coupled to higher δ^238^U_CAU_ that are up to 0.6‰ higher than inferred seawater. The approximately linear relationship in Fig. 2 is most easily interpreted in terms of the diffusion-limited effective enrichment factors (Δ^238^U_anox_) observed in modern anoxic sediments^28,57^. Figure 2 also suggests that there is a threshold, that separates these two behaviors (at Ca/U around 8 mol/µmol). None of the Site 865 data reach this threshold.

The precise reasons for variations in U concentrations at constant δ^238^U_CAU_ (horizontal array in Fig. 2) are hard to pinpoint precisely. A wide range of U concentrations have been observed in modern biogenic and bulk carbonate samples^41,42,48,58^, so it may simply be a consequence of variable mixtures of carbonate allochems and cements, despite the exclusive presence of low-Mg calcite in these samples^35–37^, such that cement bound authigenic U(VI) is diluted by low-U biogenic carbonate. Variations in grain size and surface area, and therefore faunal morphologies, are also known to affect the degree of U uptake in carbonate cements^55^. But, given other proxy evidence outlined above, one plausible explanation could be through enhanced authigenic U(IV) uptake with a δ^238^U close to seawater, as seen in some modern hypoxic to suboxic continental slope localities, where minor U enrichments are thought to occur within an effectively closed system due to extreme diffusive supply limitation^59,60^. Alternatively, minor variations in U/Ca (on the order of 0.01 µmol/mol) could reflect changes in the distribution coefficient for U into cements driven by local aqueous speciation or precipitation rate, potentially linked to ambient carbonate saturation state or pH^58^. In particular, lower pH conditions at the PETM^1,54^ could be another explanation for the minor U/Ca increase at Site 865 (Fig. 1). A final possibility is that these variations reflect the partial leaching of detrital material (Supplementary Note 2) or phosphate-associated U (which might also increase during higher productivity periods), although these should largely be avoided with the methods used here^40,41,61^. Irrespective of the exact reason for the variation in U concentrations at Site 865, Fig. 2 demonstrates that this had no resolvable influence on the δ^238^U_CAU_.

### Quantifying upper limits on the global extent of seafloor anoxia

The above discussion indicates that the Site 865 δ^238^U_CAU_ record has not been affected by local de-oxygenation or diagenesis, and likely records seawater compositions throughout the PETM. The invariance of the Site 865 record is taken to indicate the lack of a resolvable perturbation to the global U isotope mass balance during the PETM. Here we use an established biogeochemical box model^32,47^ to assess the quantitative implications of this result. We use the Site 865 δ^238^U_CAU_ dataset and higher resolution δ^13^C data from Site 401 (ref. ^1^) as targets for the biogeochemical model, which calculates the coupled dynamics of C, P, and U cycling in response to hypothesized carbon emissions. In the C–P–U model, the resultant increase in atmospheric CO_2_ and temperature drives a vegetation-mediated increase in silicate weathering which is a sink for carbon (after carbonate burial), but a source of U and P. Resultant P inputs then drive changes in global marine primary productivity and organic carbon burial, which is a sink for carbon. Burial of terrestrial organic carbon also responds directly to CO_2_ concentrations. Oxygen demand in the ocean is controlled by primary productivity and organic matter remineralization rates, and determines the extent of seafloor anoxia (where 0.25% of the modern seafloor area has <0.5 mL/L dissolved O_2_; ref. ^62^). The P cycle is also redox sensitive, accounting for the positive feedback mechanism of P recycling under anoxic conditions, which acts to further stimulate marine primary productivity. Carbon isotope mass balance tracks relative changes in sources and sinks of inorganic and organic carbon. Model U cycling and δ^238^U respond to changes in the anoxic U burial flux (scaled to benthic anoxic area) as well as weathering inputs of U, allowing us to simulate the dynamical relationship between predicted seafloor anoxia, ocean U content, and δ^238^U_sw_. Solutions are sensitive to the assumed global average Δ^238^U_anox_, where we use +0.4‰ and +0.6‰ based on modern isotopic mass balance constraints and observations of modern anoxic sediments^28,57^ (Supplementary Note 3).

**Fig. 3:**
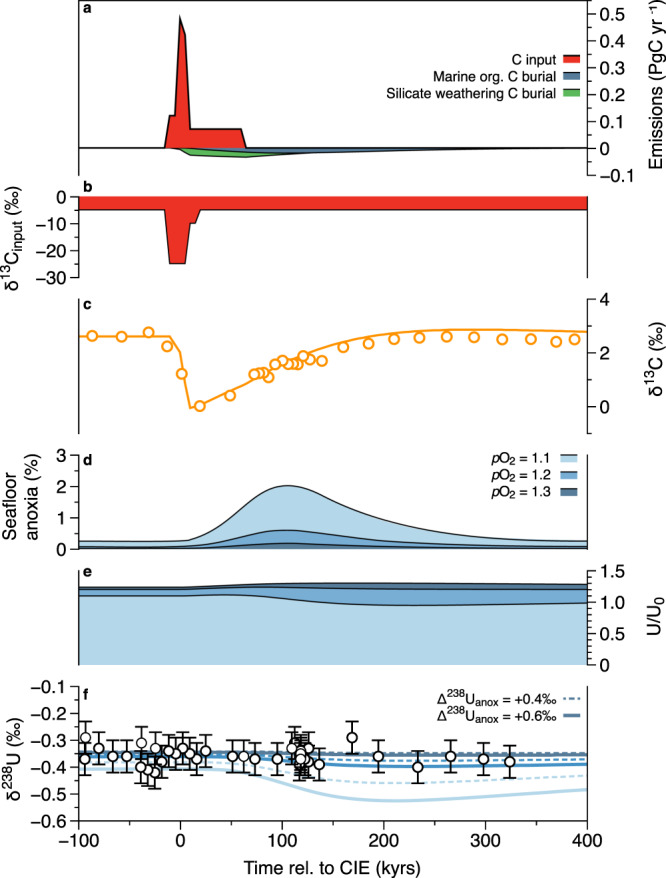
Model simulations of anoxia for PETM perturbation. Results plotted as a function of time relative to the onset of the carbon isotope excursion (CIE); **a** model forcing carbon input and calculated removal rates due to silicate weathering and marine organic carbon burial. **b** δ^13^C composition of carbon input. **c** Model δ^13^C (orange solid line) compared to smoothed and normalized planktonic foraminifera data (orange symbols) from Site 401 (ref. ^1^). **d** Seafloor anoxia estimates for model runs with *p*O_2_ = 1.1–1.3 PAL, but the same carbon emission scenario shown in **a**. **e** Calculated response of the global seawater U reservoir normalized to modern (U/U_0_) which shows an increase at the PETM onset due to increased riverine inputs. **f** Model δ^238^U_sw_ for *p*O_2_ = 1.1–1.3 PAL using Δ^238^U_anox_ = +0.6 (solid lines) and +0.4‰ (dashed lines), compared to δ^238^U_CAU_ from Site 865 (excluding anomalously low data point). Error bars for δ^238^U_CAU_ (**f**) represent the external reproducibility of the NIST-SRM 1d limestone standard (±0.06‰, 2 SD).

The model is set up for Late Paleocene background conditions (see “Methods”) and is perturbed by a plausible PETM carbon injection with an emission rate, magnitude and isotope composition (δ^13^C_input_) informed by previous studies^1–3^. In the C–P–U model, oceanic anoxia is determined by longer-term feedback processes (silicate weathering and seawater P concentrations) with a response time (>10 kyrs) on the order of the P residence time in the global ocean. The model does not account for temperature-related O_2_ solubility effects, which are deemed less important for driving OAEs^63^, or spatially controlled factors that could drive local anoxia on shorter timeframes. As such, the model estimates of global seafloor anoxia are insensitive to the precise rate and structure of carbon emission for a given total magnitude (Supplementary Note 4). But, oceanic anoxia is sensitive to background atmospheric *p*O_2_, and previous studies suggest higher than modern levels during the Paleocene^38^. Here we use *p*O_2_ = 1.1−1.3 present atmospheric levels (PAL) as a starting point. The total emission magnitude was tuned (to 9600 PgC) such that the calculated δ^238^U_sw_ response for the lower Δ^238^U_anox_ set up (+0.4‰) is within the uncertainty of the Site 865 δ^238^U_CAU_ data in the most sensitive model version (*p*O_2_ = 1.1 PAL). But, for the same perturbation, the higher Δ^238^U_anox_ set up (+0.6‰) results in a larger negative δ^238^U excursion that exceeds the limits of the observations (Fig. 3). Smaller emission magnitudes (with correspondingly smaller anoxia responses) would also be compatible with the δ^238^U_CAU_ data in all *p*O_2_ setups. Larger emissions would also be consistent but only if we assume higher *p*O_2_ (<20,400 PgC when *p*O_2_ = 1.3 PAL; <15,000 PgC when *p*O_2_ = 1.2 PAL) in order to limit the absolute extent of anoxia, or use an unrealistically low Δ^238^U_anox_ to limit δ^238^U_sw_ response. But emissions above ~15,000 PgC are not supported by other proxy datasets^1–3^. With this approach, we place a maximum limit on the extent of seafloor anoxia permitted by the data, but do not attempt to refine estimates of the total emission magnitude^1–3^.

In response to the 9600 PgC emission, enhanced silicate weathering results in a ~30% increase in oceanic P inputs, and an ~10-fold relative increase in anoxic seafloor area, regardless of *p*O_2_ (Fig. 3, Table 1). In the most sensitive model set up (*p*O_2_ = 1.1 PAL), benthic anoxia could have transiently occupied ~2% of the total seafloor area while remaining within the uncertainty range of the δ^238^U_CAU_ data. Importantly, the anoxia estimate from the dynamical model allows for a transient expansion of anoxia which is not captured by steady-state isotope mass balance solutions (Table 1), highlighting the strength of coupled biogeochemical–isotope models for investigating perturbation events. This is because anoxia is coupled to the oceanic P reservoir, which has a faster response time than the U reservoir. Furthermore, the decline in the U reservoir due to anoxic burial is muted by increased U inputs from weathering (Fig. 3) that could be important for events on the scale of the PETM.

**Table 1:** Model results comparing steady-state and dynamic model solutions for anoxia and U cycle responses.

	Steady-state model^a^	Dynamic biogeochemical model^b^		
		*p*O_2_ = 1.1	*p*O_2_ = 1.2	*p*O_2_ = 1.3
Seafloor anoxia				
Modern	0.25%^c^	0.25% for modern setup, *p*O_2_ = 1		
pre-PETM	0.09−0.16%^d^	0.25%	0.07%	0.02%
PETM	0.39−0.71%^d^	2.03%	0.60%	0.18%
Negative δ^238^U excursion				
	0.08‰^e^	0.08−0.12‰^d^	0.02−0.03‰^d^	0.005−0.008‰^d^

^a^The steady-state model implicitly accounts for changes in *p*O_2_, by deriving an extent of anoxia consistent with pre-PETM seawater of δ^238^U = −0.36‰.

^b^Dynamic solutions are given for a fixed carbon emission of 9600 PgC.

^c^Modern seafloor anoxia constraints from ref. ^62^.

^d^Min and max estimates are for Δ^238^U_anox_ = +0.4‰ and +0.6‰ model runs.

^e^δ^238^U excursion of −0.08‰ matches the dynamic model *p*O_2_ = 1.1, Δ^238^U_anox_ = +0.4‰ scenario.

In previous model studies examining PETM emission and recovery scenarios, marine organic carbon burial fluxes were specifically imposed, or inferred using an inverse model approach, ~30 kyrs after the PETM onset in order to help explain the “rapid” termination of the PETM CIE, which cannot be fully explained by silicate weathering alone^1,3,64^. By contrast, a key feature of the C–P–U model is the mechanistic response of the P cycle (and hence C and U cycles) to global temperature changes and weathering activity (Supplementary Note 5). Thus, the model calculates changes in marine organic carbon burial fluxes (and terrestrially derived organic carbon burial), in addition to silicate weathering carbon fluxes, in an internally consistent manner. With this forward modeling approach, the ~9600 PgC input flux results in a good fit to the PETM CIE, and, more importantly, the recovery of δ^13^C is well captured (Fig. 3). The cumulative burial of marine organic carbon is ~3800 PgC under this scenario, which is within the range of published estimates^1,3,64^, but with lower burial rates (<0.02 PgC/yr) spread over the extended PETM and recovery interval (Fig. 3). In comparison, silicate weathering accounts for a larger proportion of carbon burial, at ~4900 PgC. Together, these diverse model approaches highlight the importance of both silicate weathering and marine organic carbon burial in driving the recovery from the PETM.

## Discussion

Uranium isotope records in carbonate sediments can provide unique insights into the timing and severity of both local and global-scale changes in ocean de-oxygenation, depending on the oceanographic setting of the sample locality. Site 690 and 401 demonstrate the utility of δ^238^U_CAU_ as a local redox indicator in carbonate sediments, one that likely integrates bottom-water and pore-water conditions. When interpreted within a wider geochemical and paleontological framework, these data can provide information on subtle changes at and below the water sediment interface. For Sites 401 and 690, δ^238^U_CAU_ indicates the presence of anoxia, at least in pore-waters, and benthic faunal patterns suggest low-O_2_ bottom waters^21,56^ (e.g. buliminids and bolvinids survive with <0.1 to 3 mL/L dissolved O_2_ in modern settings^65^). Conditions appear to be more severely, or more permanently, reducing at Site 690 during the PETM recovery interval, where δ^238^U_CAU_ reach values similar to modern anoxic or euxinic environments^28,57^. But the continued presence of low-O_2_ tolerant foraminifera^21^ suggests either temporally fluctuating conditions, or extremely low dissolved oxygen, and the preservation of marine barite implies a lack of euxinic pore-waters^9^. The initial rise in U/Ca at Site 690 suggests that de-oxygenation may have begun ~100 kyrs before the PETM CIE, which could be an expression of background variability at the locality given that the abundance of buliminids also varies deeper in the core^21^ (Supplementary Fig. 2), or an indicator of early environmental change related to the PETM hyperthermal. Unfortunately, for Site 401, we cannot precisely constrain the timing or severity of local redox changes during the initial phase of the CIE (including the acidification interval) using U/Ca and δ^238^U_CAU_. But, U enrichment factors, measured on bulk sediment digests that were sampled at higher resolution^13^, and foraminiferal chromium isotope data^20^ suggest a brief interval of relatively more reducing conditions at the PETM onset compared to the rest of the measured interval (Supplementary Fig. 3).

In contrast to Sites 690 and 401, Site 865 remained predominantly oxygenated throughout the PETM with no evidence for the modification of δ^238^U_CAU_ by authigenic U(IV) uptake, suggesting that this locality records changes in open ocean δ^238^U_sw_, and hence reflects changes in the global extent of seafloor anoxia. The invariance in this new δ^238^U_CAU_ dataset therefore provides robust upper limits on the degree of environmental change during the PETM, indicating that any oceanic de-oxygenation must have been less than that required to perturb the global U isotopic mass balance. This result is in contrast to interpretations of published barite S isotope data in terms of a perturbation of the global S-cycle related to the expansion of sulfidic waters during the PETM^27^. The unexpected decoupling of these proxy records requires further investigation. The barite S isotope record is hypothesized to reflect an increase in water column microbial sulfate reduction that generated a transient seawater sulfide reservoir within an oxygen minimum zone (OMZ), rather than sub-sediment processes that lead to pyrite burial and S removal^27,66^. Under this scenario, U reduction would occur in a much smaller area where the OMZ impinged on the seafloor, whilst more extensive sulfate reduction could occur in open waters. The different responses might also imply an even lower sulfate reservoir than previously assumed (5 mM^27^), one that would more easily see a perturbation of S isotope mass balance.

Based on the invariance of the Site 865 record, we estimate a maximum ~10-fold relative increase in anoxic U sinks across the PETM, with anoxia likely restricted to <2% of seafloor area. Anoxia expansion above this estimate would require the effective isotope enrichment factor to be less than +0.4‰, which is inconsistent with observations from modern anoxic settings^28^. Any expansion of anoxic conditions would be accompanied by an expansion of low-O_2_ (i.e. suboxic) conditions (e.g. ref. ^20^), which would be associated with only minor U isotope fractionation and a lesser degree of authigenic U enrichment^28,59,60^ and therefore still compatible with the δ^238^U_CAU_ invariance observed at Site 865.

The model approach implicitly assumes that enhanced nutrient inputs were the primary driver of oceanic anoxia on a global scale, in a manner that is consistent with previous model results for Mesozoic OAEs^32,63^, model-data misfits for the PETM^24^, and proxy evidence in some regions of the ocean^7^. But the limits provided by U isotopes stand independent of this assumption. The extent of seafloor anoxia during the PETM may have been an order of magnitude lower than the most severe of the Mesozoic OAEs; OAE 2 (ref. ^32^). This difference, within the framework presented here, is mainly due to the shorter duration of emission, but with comparable rates (i.e. smaller total emission magnitude), and higher background *p*O_2_ for the PETM. In reality, local scale factors, that are not accounted for in the C–P–U model, will also play an important role (such as basin configuration and temperature controlled O_2_ solubility) and in many localities peak anoxia occurred immediately after the onset of the PETM CIE (e.g. ref. ^12^) rather than the later global maximum that is predicted by the C–P–U model. This model prediction is, however, consistent with the timing of the second episode of de-oxygenation at Site 690 during the PETM recovery interval, which is thought to be driven by weathering changes in Antarctica and resultant eutrophication^10,13,67^. These differences highlight the relative sensitivities of some regions to global climate change based on the paleogeographic and oceanographic setting, but also the prominent role of silicate weathering and marine organic carbon burial, especially during the recovery interval. The longer term and global perspectives afforded by U isotopes therefore offer important constraints that can be used in combination with local proxy records and spatially resolved models to better understand environmental change in a consistent manner for a variety of OAEs.

## Methods

### Chemical preparation

Samples were processed and measured at ETH Zurich (ETHZ; Sites 865, 401, and 690) and the Open University (OU; Site 865). At ETHZ, ~600 mg of dry sample powder was leached using 2 × 40 mL of 1 M ammonium acetate (pH 5) for 24 h at room temperature in order to selectively dissolve the bulk carbonate fraction^40^. The excess volume of acid ensures near complete carbonate dissolution with this method. At the OU, selective carbonate leaching was performed using 0.5 M acetic acid and dissolved ~50% of the carbonate. The IRMM-3636 U double spike was added before column chemistry for all samples.

At ETHZ, leachates were oxidized and dried down with excess concentrated HNO_3_. The salt precipitates were then converted to chloride form with 7 M HCl, dried and re-dissolved in 10 mL of 1 M HCl. The solution was loaded onto ~0.2 mL of 50–100 µm pre-cleaned RE-Resin (Triskem Technologies). Columns and resin were pre-cleaned with 2 mL of 0.2 M HCl + 0.3 M HF, rinsed with MilliQ water, and pre-conditioned with 2 × 2 mL of 1 M HCl. Matrix elements were eluted during the first column pass using 2 mL of 1 M HCl and U collected using 2 mL of 0.2 M HCl + 0.3 M HF. The samples were dried down and re-dissolved in 1 mL of 1 M HCl for a second column pass on a fresh column. Residual matrix was eluted on the second pass using 4 mL of 1 M HCl followed by 2 mL of 0.2 M HCl and U was collected using 2 mL of 0.2 M HCl + 0.3 M HF. Before analysis, samples were vigorously oxidized overnight with concentrated H_2_O_2_ and HNO_3_ in order to break down minor resin contribution, and re-dissolved in 0.2 M HCl. Total procedural blanks are estimated at ~<30 pg for U.

At the OU, leachates were similarly oxidized and dried down with excess concentrated HNO_3_. The samples were then taken up in 7.5 M HNO_3_ and loaded on a 2-mL pre-column filled with Bio-rad AG1X8 resin. The U fraction was eluted with 0.5 M HCl, before being dried down and taken up in 3 M HNO_3_ for loading onto a 0.2-mL column packed with UTEVA resin (Triskem Technologies). The remaining matrix was eluted with 5 M HCl before the U fraction was collected using 0.5 M HCl. Similarly to the ETHZ protocol, samples were oxidized overnight with concentrated H_2_O_2_ and HNO_3_ before being taken up in 2% HNO_3_ for MC-ICPMS measurements. Total procedural blanks measured by isotope dilution were between 6 and 15 pg of U.

### Trace and major element concentrations

Leachates were directly aliquoted and diluted 200–400 times in 2% HNO_3_. Major and trace elements were measured using a Thermo–Finnigan Element XR ICP–MS with an internal indium (In) standard and a blank correction applied. Concentrations were calculated relative to an in-house, well characterized and gravimetrically produced artificial standard with matrix characteristics similar to carbonates and reported normalized to Ca (µmol/mol). U/Ca are not reported for OU samples as they represent incomplete carbonate dissolution. Uncertainties on metal concentrations and ratios are twice the relative standard deviation (RSD) of a carbonate standard which are typically between 10 and 15%.

### Isotope measurements

Isotope ratios were measured on a Neptune Plus (Thermo– Finnigan) MC–ICPMS equipped with an Aridus II DSN (CETAC) and using a PFA nebulizer and spray chamber (CPI) sample introduction system at ETHZ or OU. Given the low concentrations in the leachates “jet + X-cones” were used. Uranium isotope ratios are reported relative to the standard CRM-145 = 0‰ for ^238^U/^235^U and secular equilibrium for ^234^U/^238^U, and presented as:

1 $${\updelta}^{238} {\mathrm{U}} = \left[ \left( {\,\!}^{238}{\mathrm{U}}/{\!\,}^{235}{\mathrm{U}}_{{\mathrm{sample}}} \right)/\left( {\!\,}^{238}{\mathrm{U}}/{\!\,}^{235}{\mathrm{U}}_{{\mathrm{CRM}}145} \right) - 1 \right] \times 1000,$$

2 $$\delta ^{234}{\mathrm{U}} = \left[ \left( {\,\!}^{234}{\mathrm{U}}/{\,\!}^{238}{\mathrm{U}}_{{\mathrm{sample}}} \right)/\left( {\,\!}^{234}{\mathrm{U}}/{\,\!}^{238}{\mathrm{U}}_{{\mathrm{sec}}.\,{\mathrm{eq}}} \right) - 1 \right] \times 1000.$$

Internal errors (2 SE) for δ^238^U measurements were typically 0.02–0.04‰ at ion beam intensities of ~35 to 40 V (using 10^11^ ohm resistors) for ~40 p.p.b. solutions. At ETH, we used two secondary standards to assess external reproducibility and accuracy. First, a uraninite standard, CZ-1, used previously at ETH Zürich, was run between every five unknown samples and gives a δ^238^U of −0.04 ± 0.05‰ (*n* = 68, 2 SD) during the measurement period, which is identical to values reported elsewhere^29,40,60^. To determine the external reproducibility of a carbonate sample, we used the NIST SRM-1d argillaceous limestone that was processed in two sample batches in the same manner as samples, but dissolved using 1 M HCl. NIST SRM-1d gave a δ^238^U of −0.12 ± 0.06‰ (2 SD, *n* = 14) which is identical to values reported elsewhere^43^. At the OU, SRM 950A was run between every three unknown samples and gives, over the analysis period, a δ^238^U of 0.03 ± 0.03‰ (*n* = 8, 2 SD), similar to published values^28^. Additionally, two natural standards, processed through column chemistry, were run for assessing accuracy and long-term reproducibility: Seawater, yielding a δ^238^U value of −0.39 ± 0.05‰ (*n* = 8, 2 SD), similar to published values (−0.39 ± 0.01‰)^28^; BE-N basalt powder yielding a δ^238^U of −0.30 ± 0.03‰ (*n* = 7, 2 SD), within error of published values (−0.33 ± 0.03‰)^59^.

### Age model

Here we use the ^3^He age model^68^ rather than the cyclostratigraphic ages^69^ for all sites. The ^3^He model is favored because of the consistency of de-oxygenation trends in δ^238^U_CAU_ with barium isotope and BAR records at Site 690 when using this age model^10^ (Supplementary Fig. 2; Supplementary Note 2). We use age tie points for the CIE onset from Röhl et al.^69^ as used for Sites 401 and 690 in previous studies^1^. The age model for Site 865 is less well constrained compared to the Site 690 and 401 records. Here we match the foraminiferal δ^13^C data from Site 865 to the bulk carbonate record for Site 690, and check this against planktic records from Site 401, as shown in Supplementary Fig. 4. There is a small gap in the Site 865 record that is thought to be caused by winnowing. This has little bearing on the completeness of the δ^238^U_CAU_ record and subsequent interpretations as the U isotope response to the PETM perturbation is expected to be preserved later due to the non-linearities of anoxia and the large seawater U reservoir (Fig. 3). The uncertainties in the age model have no significant bearing on the findings of anoxia (Supplementary Note 4).

### Dynamic biogeochemical model

The C–P–U model is informed by previously established biogeochemical models (COPSE^38^) and calculates the coupled dynamics of C, P, and U cycling associated with changes in temperature, weathering, and oceanic anoxia, in response to hypothesized CO_2_ perturbations. Full model details are given in ref. ^32^. We make two changes to the model described there: (1) We do not account for changes in O_2_ due to changes in the burial of organic carbon (because the timescale is too short for significant changes in O_2_ and we want to treat O_2_ as an uncertain boundary condition); (2) We increase the climate sensitivity to doubling atmospheric pCO_2_ to a value of ~5 °C, appropriate for the PETM^60,70^ in order to better capture the weathering response to CO_2_ perturbation at this time.

The model is set up with appropriate boundary conditions for background degassing (D) uplift and erosion (E) and weatherability (W) based on COPSE estimates^38^. With the revised climate sensitivity, these baseline settings result in atmospheric pCO_2_ of 1.65 PAL (PAL = ~300 p.p.m.), in reasonable agreement with constraints from other PETM studies^3^. We use a total C emission scenario informed by previous estimates^1–3^ with a total duration of 75 kyrs. Peak carbon inputs reach 0.54 PgC/yr and are followed by a prolonged C leak at a rate of 0.07 PgC/yr. The isotopic composition of the C source is varied between −5 and −25‰, representing a mix of C from mantle sources and organic C reservoirs (e.g. methane or oxidized organic C), in order to fit as well as possible the planktic δ^13^C record from Site 401^1^. The δ^13^C of organic carbon (δ^13^C_org_) is assumed to be −25‰ throughout for simplicity.

### U isotopic mass balance

The U-cycle model is as presented in ref. ^32^. It assumes that the riverine input of U is driven by silicate weathering (*F*_w_), all U sinks scale linearly with U concentration, the hydrothermal sink of U additionally scales with degassing from seafloor spreading (*D*), and the anoxic sink of U also scales with *f*_anoxic_, the fraction of anoxic seafloor:

3 $${\mathrm{d}}U/{\mathrm{d}}t = {{F}}_{{\mathrm{riv}}}-{{F}}_{{\mathrm{hyd}}}-{{F}}_{{\mathrm{anoxic}}}-{{F}}_{{\mathrm{other}}},$$

4 $${{F}}_{{\mathrm{riv}}} = {{k}}_{{\mathrm{riv}}} \times {{F}}_{\mathrm{w}}/{{F}}_{{\mathrm{w0}}},$$

5 $${{F}}_{{\mathrm{hyd}}} = {{k}}_{{\mathrm{hyd}}} \times {{D}} \times \left( {{{U}}/{{U}}_0} \right),$$

6 $${{F}}_{{\mathrm{anoxic}}} = {{k}}_{{\mathrm{anoxic}}} \times \left( {{{U}}/{{U}}_0} \right) \times {{f}}_{{\mathrm{anoxic}}}/{{f}}_{{\mathrm{anoxic}}0},$$

7 $${{F}}_{{\mathrm{other}}} = {{k}}_{{\mathrm{other}}} \times \left( {{{U}}/{{U}}_0} \right),$$

where the subscript “0” denotes present day values. For a modern *U*_0_ = 1.85 × 10^13^ mol, we use estimates of *k*_anoxic_ = 6.2 × 10^6^ mol/yr, *k*_hyd_ = 5.7 × 10^6^ mol/yr, *k*_other_ = 36 × 10^6^ mol/yr, and therefore *k*_riv_ = 47.9 × 10^6^ mol/yr. The corresponding isotope mass balance is:

8 $${\mathrm{d}}({{U}} \times {\delta}_{\mathrm{U}})/{\mathrm{d}}t = 	\, {{F}}_{{\mathrm{riv}}} \times {\delta}_{{\mathrm{riv}}}-{{F}}_{{\mathrm{hyd}}} \times ({\delta}_{\mathrm{U}} + {\Delta} _{{\mathrm{hyd}}}) -{{F}}_{{\mathrm{anoxic}}} \times ({\delta}_{\mathrm{U}} + {\Delta} _{{\mathrm{anoxic}}})\\ \,	 -{{F}}_{{\mathrm{other}}} \times ({\delta}_{\mathrm{U}} + {\Delta} _{{\mathrm{other}}}).$$

From using the chain rule:

9 $${\mathrm{d}}\delta _{\mathrm{U}}/{\mathrm{d}}t = ({{F}}_{{\mathrm{riv}}} \times(\delta _{{\mathrm{riv}}}-\delta _{\mathrm{U}})-{{F}}_{{\mathrm{hyd}}} \times {\Delta} _{{\mathrm{hyd}}}-{{F}}_{{\mathrm{anoxic}}} \times {\Delta} _{{\mathrm{anoxic}}}-{{F}}_{{\mathrm{other}}} \times {\Delta} _{{\mathrm{other}}})/{{U}}.$$

For the modern, we assume *δ*_riv_ = −0.29‰, Δ_hyd_ = 0.2‰, Δ_anoxic_ = 0.5‰, and *δ*_U_ = −0.39‰, requiring Δ_other_ = 0.0153‰ for steady state. Solving for steady state and rearranging gives:

10 $$\frac{{{{f}}_{{\mathrm{anoxic}}}}}{{{{f}}_{{\mathrm{anoxic}}0}}} = \frac{{{\delta }}_{{\mathrm{riv}}} \times \left( {{{k}}_{{\mathrm{other}}} + {{k}}_{{\mathrm{hyd}}} \times {{D}}} \right) - \left( {{\delta }}_{\mathrm{U}} + {\Delta} _{{\mathrm{hyd}}} \right) \times {{k}}_{{\mathrm{hyd}}} \times {{D}} - \left( {{\delta }}_{\mathrm{U}} + {\Delta} _{{\mathrm{other}}} \right) \times {{k}}_{{\mathrm{other}}}}{\left( {{\delta }}_{\mathrm{U}} + {\Delta} _{{\mathrm{anoxic}}} - {\delta}_{{\mathrm{riv}}} \right) \times {{k}}_{{\mathrm{anoxic}}}}.$$

The present-day anoxic fraction (*f*_anoxic0_) is estimated to be 0.2–0.3% of seafloor area^62^. In the full model, *f*_anoxic0_ = 0.002473 in the middle of this range. Equation (9) allows us to derive steady-state anoxic fraction for a given set of parameters and seawater *δ*_U_. At 55 Ma, we assume no change in *δ*_riv_ = −0.29‰, Δ_hyd_ = 0.2‰, Δ_other_ = 0.0153‰. We assume elevated degassing *D* = 1.4 (ref. ^38^) and explore Δ_anoxic_ = 0.4‰ as well as 0.6‰, for a pre-PETM seawater of *δ*_U_ = −0.36‰. In the steady-state solution, an excursion magnitude of −0.08‰ is defined by the dynamic model for *p*O_2_ = 1.1, Δ_anoxic_ = 0.4‰ scenario, in order to compare the two approaches.

## Supplementary information

Supplementary Information

## Data Availability

All data generated or analyzed during this study are included in this published article (and its Supplementary information files). Source data are provided with this paper.

## Source data

Source Data
