# Supplementary material for: Upper limits on the extent of seafloor anoxia during the PETM from uranium isotopes
Source: Nat Commun. 2021 Jan 15;12:399. doi: 10.1038/s41467-020-20486-5 (PMC7810695; doi:10.1038/s41467-020-20486-5)
Supplement: Supplementary file 1 — Supplementary Information [file 41467_2020_20486_MOESM1_ESM.pdf]

Supplementary Information for 'Upper limits on the extent of seafloor anoxia during the PETM from uranium isotopes' by Clarkson et al.

Supplementary Note 1

For Site 690, suboxic bottom water conditions were previously inferred using U and Mn enrichment factors (EFs)[1], and the new U/Ca and $\delta^{238}U_{CAU}$ datasets generally agree with these findings but show a more detailed pattern of de-oxygenation. The initial rise in U/Ca, coupled to $\delta^{238}U_{CAU}$, at Site 690 are not seen in U EFs but could reflect either pre-PETM events or an expression of de-oxygenation at the PETM itself but recorded in the underlying sediment. The former scenario could be consistent with the initial rise in bolvinids, but the latter scenario is more consistent with the timing of increased abundances of buliminid foraminifera and a marked decrease in the oligotrophic indicator species *N.truempyi*[2] (Supplementary Figure 2). The later rise in $\delta^{238}U_{CAU}$ occurs during the PETM recovery interval and corresponds to increased barium and barite accumulation rates[3] (using the ^{3}He age model[4]; Supplementary Figure 2), high coccolithophore production and an increase in kaolinite clays that marks enhanced erosion of Antarctica[5,6]. Together, these trends support previous interpretations that eutrophication drove bottom water de-oxygenation in the southern Atlantic Ocean during the PETM recovery period[1]. The magnitude of U isotope fractionation recorded in these samples are compatible with modern anoxic or euxinic environments, although the total U enrichments are relatively low for such settings[7-13] and there is no distinct organic carbon accumulation during the recovery interval. The preservation of barite[14] argues against the development of euxinic conditions. Moreover, the presence of benthic fauna throughout the studied interval argues against sustained bottom water anoxia, suggesting that Site 690 was characterized by anoxic pore-waters and predominantly sub-oxic or only temporarily anoxic bottom waters.

At Site 401 the U/Ca and $\delta^{238}U_{CAU}$ increase is associated with a general decline in ostracod diversity[15] which directly correspond to Cr isotope and Ce anomaly evidence for de-oxygenation[16] (Supplementary Figure 3). The Ce anomaly results were interpreted to reflect anoxic pore-waters at Site 401 at the very onset of the PETM[16], and the magnitude of the

$\delta^{238}U_{CAU}$ increase is consistent with sub-oxic to anoxic pore-waters and low-O_2 (sub-oxic) bottom waters that persisted into the beginning of the PETM recovery interval. But the magnitude of $\delta^{238}U_{CAU}$ increase suggests that conditions were less reducing, or less sustained, at Site 401 than Site 690. Trends in U/Ca and $\delta^{238}U_{CAU}$ generally reflect trends in U EFs[1] (Fig. S2), but are better able to distinguish the onset the de-oxygenation from a pre-PETM background, partly due to sample coverage. The development of reducing conditions at Site 401 is plausibly related to heightened productivity as a result of an increased flux of weathering products to the North Atlantic[17,18].

Previous geochemical studies on the redox evolution at Site 865 are limited but benthic foraminifera show an increased abundance of buliminid and bolvinid species coincident with the U/Ca observed here[19] (Supplementary Figure 1), similar to the trends at Site 690. Additional support comes from studies of the nearby Site 1209 at Shatsky Rise, which is a similar depositional and oceanographic setting. Site 1209 consistently has Ce/Ce* < 0.5 that are suggestive of oxygenated bottom and pore-waters throughout the PETM[16], but benthic fauna abundances are interpreted to reflect a temporary de-oxygenation event[20] in a similar manner Site 865 (ref. 19) (Supplementary Figure 1), suggesting regionally extensive environmental deterioration. Despite this, U EFs at Site 1209 do not show any systematic change across the PETM although raw U concentrations do show a minor increase coincident with benthic faunal turnover[1]. The lack of clear U enrichment in U EFs could be due to the low degree of U enrichment relative to detrital estimates, whereas a more consistent picture is gained from using U/Ca of carbonate leachates at Site 865, suggesting that the reliance of detrital normalization to calculate EFs might obscure subtler redox trends. Moreover, the U EF were calculated using the average U concentrations in the upper continental crust, which would over-estimate the detrital correction given that the detrital phases of pelagic sediments are typically depleted in U compared to the UCC[21,22]

Supplementary Note 2

Whilst the leaching technique employed here largely avoids detrital components[21], there is a reasonable correlation of increasing U/Ca with decreasing Ca concentrations at Site 865 (r^2=0.51) (Fig. S5). But this could simply reflect the co-occurrence of de-oxygenation with the lower $CaCO_3$ interval at the PETM, or potentially the dilution of higher U/Ca cements by low U/CA biogenic calcite. Furthermore, Site 865 shows only limited changes in Al or Ti/Ca, which do not appear correlated with U/Ca (Supplementary Figure 5). Stronger correlations are seen for Ba/Ca with U/Ca (Supplementary Figure 5) which could be supporting evidence for heightened productivity and potentially lower O_2 conditions. Some, but not all, of the Site 865 samples also show higher Sr/Ca with higher U/Ca, which again could support the interpretation of heightened productivity.

Higher U/Ca correlate with lower Ca concentrations at Site 401 (Supplementary Figure 5) reflecting a secular decrease in $CaCO_3$ across the PETM (Supplementary Figure 3), and also higher Ti/Ca and Al/Ca that were likely a consequence of increased hinterland erosion during the PETM hyperthermal[17,18]. But, enrichments in U are clearly evident above the trends with detrital indicators and accompanied by higher $\delta^{238}U_{CAU}$ values, identifying that U enrichments from de-oxygenation occur superimposed on a secular $CaCO_3$ decrease (Supplementary Figure 3). Furthermore the rise in U/Ca at the PETM for Site 401 is also visible in U EFs from bulk digests, that correct for detrital contributions[1]. It is therefore unlikely that detrital leaching itself controls the main U/Ca increases here, but could partly explain the differences in background U/Ca compared to Site 865. The generally higher Ti concentrations at Site 401, could indicate that the detrital phases are more easily leached than the other localities, in addition to generally lower % $CaCO_3$. Indeed, Site 401 also shows highly elevated $\delta^{234}U$ values compared to the other locations (see Data Tables), implying greater liberation of ^{234}U from damaged lattice sites

during leaching. Similarly to Site 865, Ba/Ca and Sr/Ca show a good positive correlation with U/Ca, potentially supporting the interpretation of increased productivity as a driver of U enrichments.

At Site 690 there is no indication of heightened detrital contributions from Al or Ti that correspond to U enrichments, and the highest U/Ca occur in the samples with highest Ca concentration. This reflects the fact that productivity at Site 690 appears to be driven by high carbonate (coccolithophore) production (Supplementary Figure 2). The same major U enrichments during the PETM recovery interval are seen in U EFs [1], but these do not clearly resolve the first increase in U/Ca before the PETM, that correlate with $\delta^{238}U_{CAU}$. This is likely because the degree of U enrichment is too low to be resolvable over contributions from detrital components, but the carbonate leachate avoids these detrital phases and so is better able to identify lesser U enrichments.

Supplementary Note 3

The relationship of oceanic anoxia to $\delta^{238}U_{sw}$ is dependent on the effective enrichment factor for U(IV) incorporation into reducing sediments ($\Delta^{238}U_{anox}$). In the modern ocean, this can vary depending on numerous local factors such as the degree of bottom water de-oxygenation, basin restriction, organic carbon loading, sedimentation rates and diffusion limitations[7,23]. As such, there is a large uncertainty in this value for studies of past oceanic anoxia. Previous studies often extrapolate from a subset of anoxic basins[24,25], or rely on the observed offset between carbonates and anoxic shales for a given time period[26]. Here we use the average modern day value that satisfies mass balance constraints (+0.4‰), and the upper value associated with diffusion limited U reduction in anoxic sediments (+0.6‰), which are also indicated by the $\delta^{238}U_{CAU}$ data from sites 401 and 690. Whilst these estimates are low compared with other

mass-balance models[27,28] , we recognize that a larger $\Delta^{238}U_{anox}$ would result in a larger $\delta^{238}U_{carb}$ excursion and hence require a lower degree of anoxia for the perturbation scenario. Thus, our approach is in-keeping with the aim of examining the maximum extent of anoxia permissible by the data.

Supplementary Note 4

The results of the biogeochemical model are deemed insensitive to the range of age uncertainty between current age models, as total seafloor anoxia is a response to long-term (>50 kyr) processes (i.e. weathering and nutrient supply). Similarly, anoxia and modeled $\delta^{238}U$ should be relatively insensitive to the precise C emission rate. To test this assumption, the total C emission of 9600 PgC was released i) equally distributed across 75kyr and ii) in a single 5kyr pulse for the O_{2atm}=1.1, $\Delta^{238}U_{anox}$ =+0.4‰ model. The size of the resulting $\delta^{238}U$ excursions are 0.077‰ and 0.083‰, with seafloor anoxia of 2.03% and 2.23% respectively. In comparison, the preferred 'peak +leak' emission structure constrained by previous works[29,30] results in the same excursion magnitude as scenario i) with a 0.077‰ $\delta^{238}U$ excursion and 2.03% anoxia. The small differences are mainly due to the competing effect of C burial processes in the longer release scenario that dampen the temperature increase, and hence total weathering response and P inputs. This illustrates that modelled seafloor anoxia is largely a function of the total C emission size rather than a precise rate of emission, unlike model results for ocean pH or $\delta^{13}C$ (ref. 29).

Supplementary Note 5

The model is clearly sensitive to the formulation of the functions describing the carbon cycle, climate sensitivity, weathering and phosphorus cycling, and ocean anoxia. But, the forward model represents an internally consistent, alternative approach to previous model attempts

which results in an excellent fit for the PETM $\delta^{13}C$ recovery. A particular limitation is that the C-P-U model does not consider non-steady-state behavior of the ocean carbonate system on 10 kyr timescales. Hence, it underestimates the initial increase in pCO_2 (0.52 PAL increase) and global temperature (~2°C increase) at the PETM and may underestimate the weathering and anoxia responses. Our focus here is on setting some bound on the expansion of anoxia consistent with little change in $\delta^{238}U$, given a prescribed perturbation, and thus this limitation is not critical. A larger modelled temperature increase would require a less sensitive weathering function to maintain lower anoxia within the limits of the $\delta^{238}U_{CAU}$ data. Thus, it would lean the results even further towards high atmospheric pO_2 and low background anoxia as the most reasonable pre-PETM boundary conditions.

SUPPLEMENTARY FIGURES

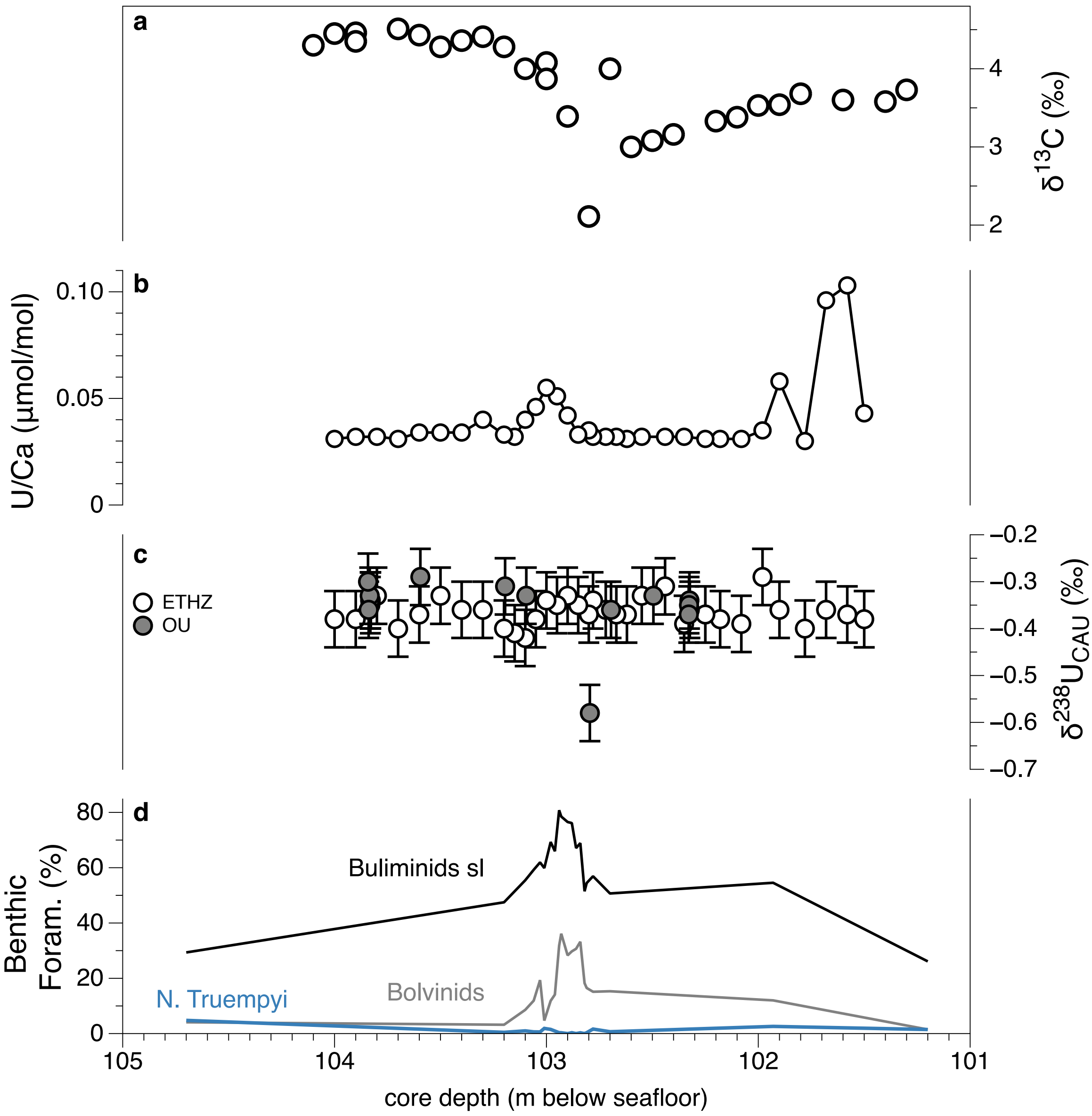

Supplementary Figure 1. Contextual accompanying data for Site 865. **a** $\delta^{13}C$ data for Valescoensis foraminifera from ref. 31 clearly resolve the PETM CIE. **b** U/Ca and **c** $\delta^{238}U$ from this study. **d** Benthic foraminiferal abundances from ref. 19 demonstrate an increase in low-O_2 tolerant species co-eval with the U/Ca increase around the onset of the PETM, and accompanied by a decrease in the oligotropic indicator species N. Truempyi. Error bars for $\delta^{238}U_{CAU}$ represent the external reproducibility of the NIST-SRM 1d limestone standard (±0.06‰, 2SD)

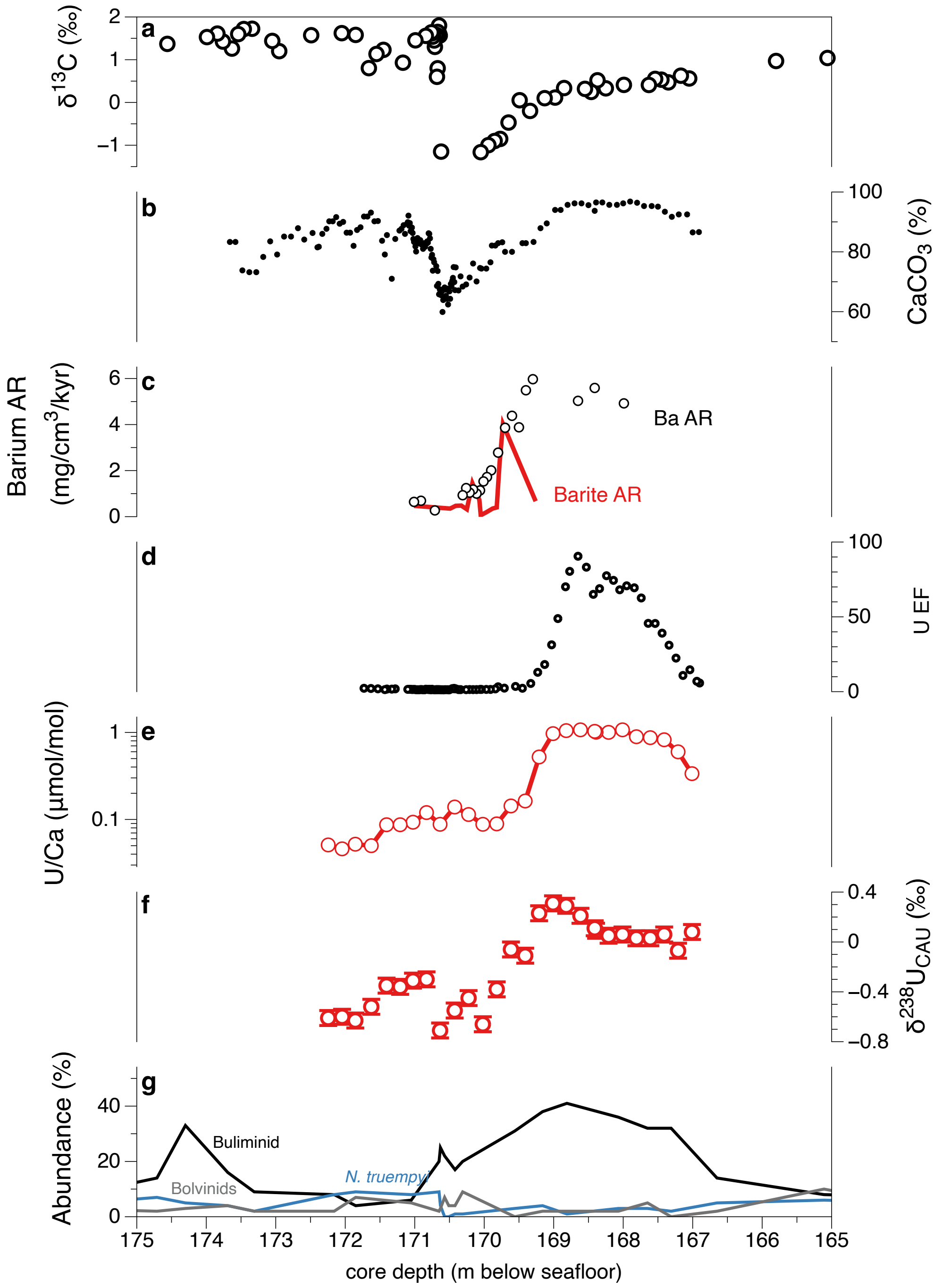

Supplementary Figure 2. Contextual and supporting proxy data from Site 690. **a** $\delta^{13}C$ data for *N.Truempyi* from compilation in ref. 32 after ref. 33-35. **b** $CaCO_3$ data from ref. 36 show high carbonate interval during the PETM recovery period that is associated with high productivity identified using Ba excess accumulation rates (AR) and barite AR[3,14] **(c)**. During this productivity episode U enrichment factors (EF) **(d)**, U/Ca **(e)** and $\delta^{238}U_{CAU}$ **(f)** indicate more reducing conditions. There is particularly clear correspondence between Ba AR (using the ^{3}He age model[3]) and $\delta^{238}U_{CAU}$ between 170–168.5m. The identification of reducing conditions using U EF[1] generally support the interpretations from U/Ca and $\delta^{238}U_{CAU}$ during the PETM recovery interval (176–

170mbsf) but earlier changes (171.5–170mbsf) are not resolved. This is likely due to the very low degree of U enrichment which is masked by detrital corrections, but identified by U/Ca and $\delta^{238}U_{CAU}$. **g** Benthic foraminifera abundances[2] show increased abundances of low-O_2 tolerant species with evidence for more reducing conditions, firstly with the initial rise of Bolvinids at ~172 mbsf when U/Ca and $\delta^{238}U_{CAU}$ increase, and then the increase in Buliminids that covers the remainder of the PETM recovery interval. Error bars for $\delta^{238}U_{CAU}$ represent the external reproducibility of the NIST-SRM 1d limestone standard (±0.06‰, 2SD)

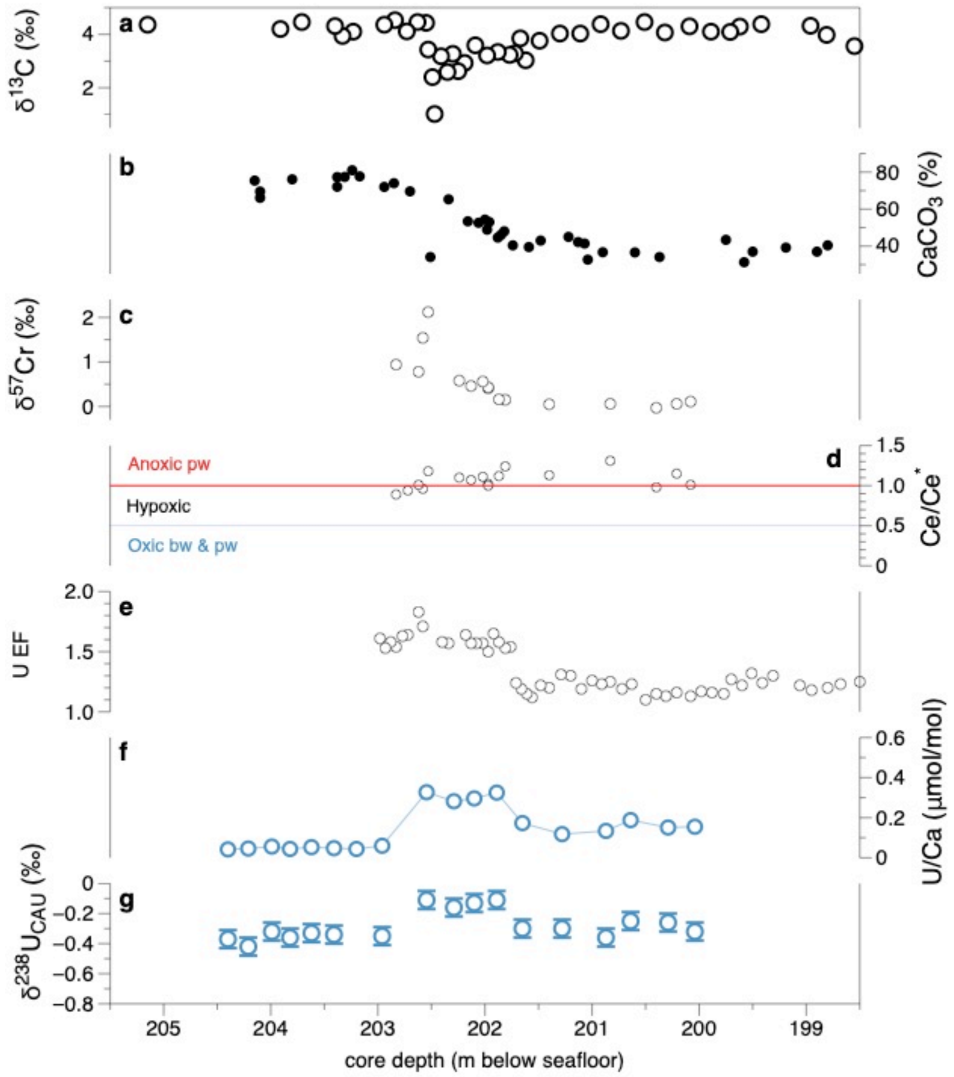

Supplementary Figure 3. Supporting datasets from Site 401. **a** $\delta^{13}C$ from ref. 29. **b** $CaCO_3$ data from ref.[1] showing a secular decrease in carbonate across the PETM that is related to increased terrigenous inputs as a result of weathering changes in the hinterland[17,18]. **c** Chromium isotope, **(d)** cerium anomaly (Ce/CE*)[16] and **(e)** U enrichment factors (U EF) [1] identify a move to more reducing conditions at the onset of the PETM which are clearly identified by U/Ca **(f)** and $\delta^{238}U_{CAU}$ **(g)**. Error bars for $\delta^{238}U_{CAU}$ represent the external reproducibility of the NIST-SRM 1d limestone standard (±0.06‰, 2SD)

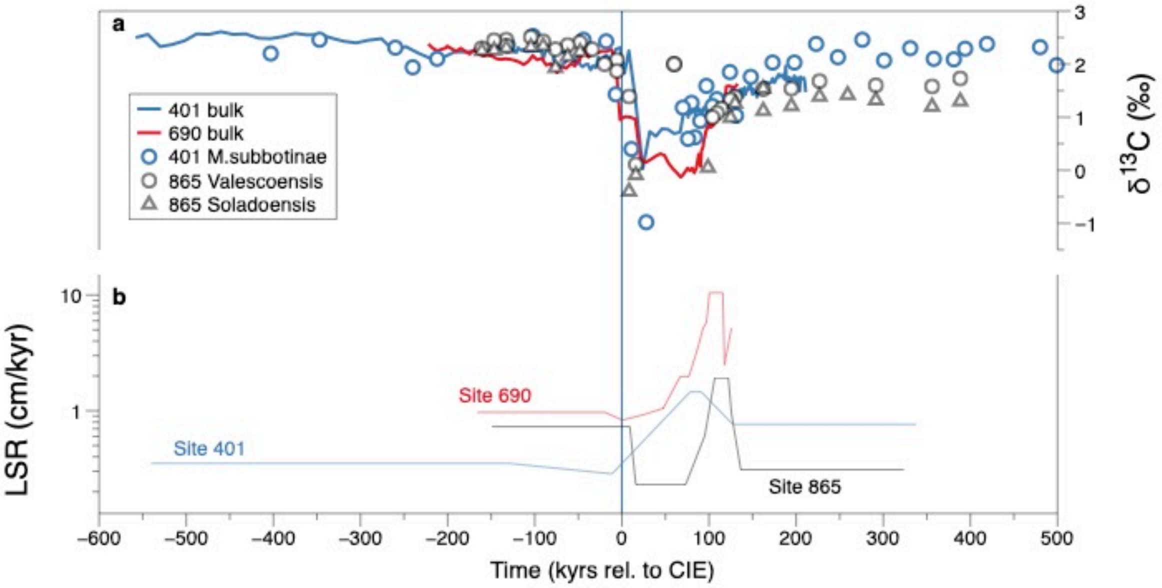

Supplementary Figure 4. a Correlations based on $\delta^{13}C$ are used to derive the age models for Site 401 and 865, using the ^{3}He age model[4] from Site 690. Site 401 and 690 age models are as reported elsewhere[29], and Site 865 foraminiferal $\delta^{13}C$ data[31] was visually matched to the Site 690 bulk record[37]. **b** Resulting Linear Sedimentation Rates (LSR) are shown for the samples used in this study.

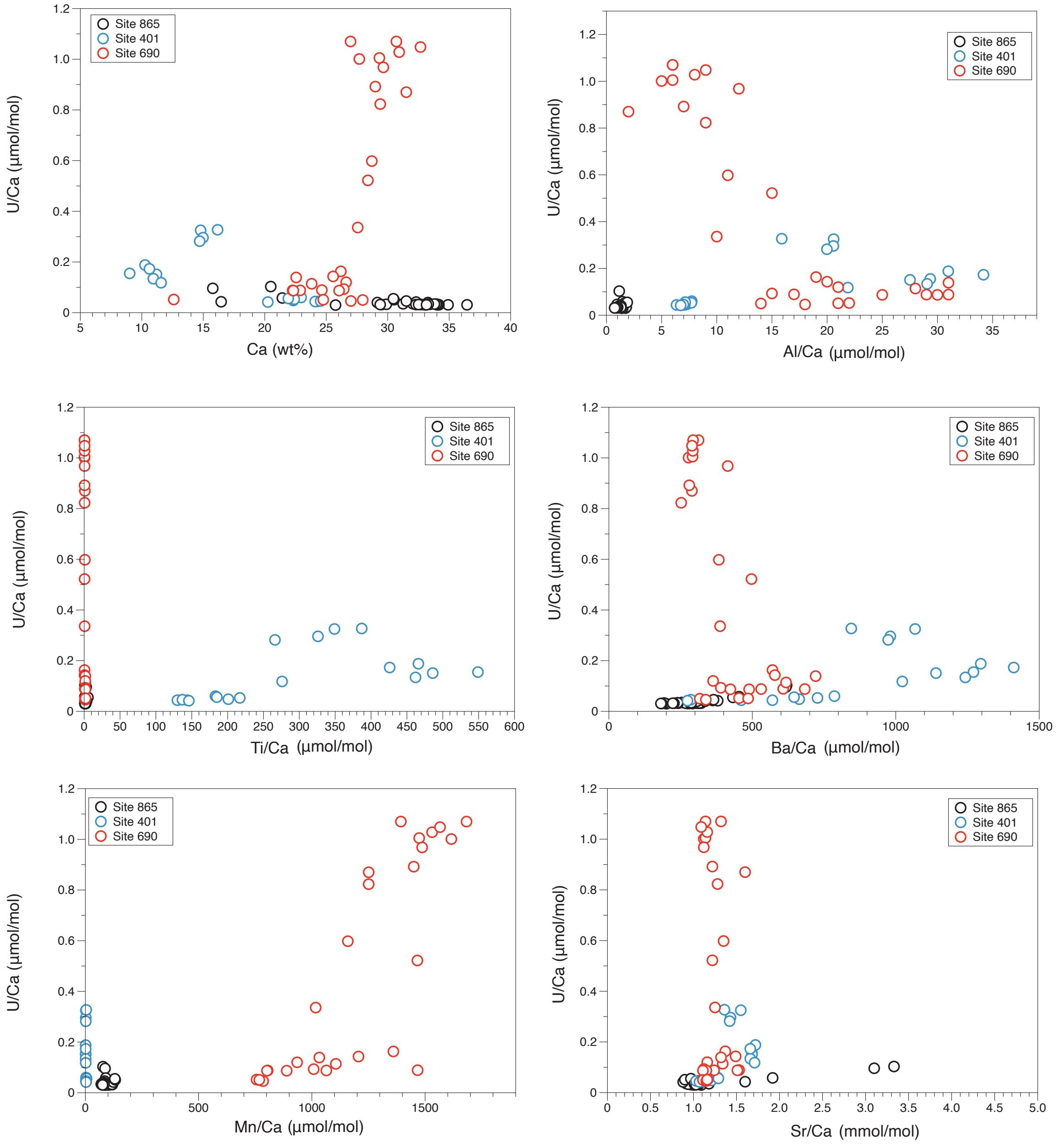

Supplementary Figure 5. U/Ca plotted as a function of detrital, productivity and diagenesis indicators. Details are discussed in Supplementary Note 1 and 2. The relationship of U/Ca with Ti/Ca and Al/Ca do not identify a clear control of detrital phases on U/Ca ratios for the three sites. A general increase in U/Ca with Ba/Ca for Site 865 and 401 support the concept of greater productivity driving locally reducing conditions. The relationship is less clear for Site 690 where the major change in sedimentation rates masks the increase in

Ba/Ca, which is more clearly seen in Ba accumulation rates (Supplementary Figure 2). The correlations of Mn/Ca and U/Ca for Site 690 suggests there is the reductive dissolution of Mn in pore-waters which are remobilized and precipitate as Mn-carbonate. Sr/Ca again can suggest a productivity control on locally reducing conditions, but the slightly lower Sr/Ca for Site 865 is likely related to diagenesis[38]

SUPPLEMENTARY REFERENCES

1 Pälike, C., Delaney, M. L. & Zachos, J. C. Deep-sea redox across the Paleocene-Eocene thermal maximum. *Geochemistry, Geophysics, Geosystems* **15**, 1038-1053 (2014).

2 Thomas, E. & Shackleton, N. J. The Paleocene-Eocene benthic foraminiferal extinction and stable isotope anomalies. *Geological Society, London, Special Publications* **101**, 401-441 (1996).

3 Bridgestock, L., Hsieh, Y.-T., Porcelli, D. & Henderson, G. M. Increased export production during recovery from the Paleocene–Eocene thermal maximum constrained by sedimentary Ba isotopes. *Earth and Planetary Science Letters* **510**, 53-63 (2019).

4 Farley, K. & Eltgroth, S. An alternative age model for the Paleocene–Eocene thermal maximum using extraterrestrial 3He. *Earth and Planetary Science Letters* **208**, 135-148 (2003).

5 Kelly, D. C., Zachos, J. C., Bralower, T. J. & Schellenberg, S. A. Enhanced terrestrial weathering/runoff and surface ocean carbonate production during the recovery stages of the Paleocene-Eocene thermal maximum. *Paleoceanography* **20** (2005).

6 Robert, C. & Kennett, J. P. Antarctic subtropical humid episode at the Paleocene-Eocene boundary: Clay-mineral evidence. *Geology* **22**, 211-214 (1994).

7 Andersen, M. *et al.* A modern framework for the interpretation of $^{238}U/^{235}U$ in studies of ancient ocean redox. *Earth and Planetary Science Letters* **400**, 184–194 (2014).

8 Andersen, M., Stirling, C. H. & Weyer, S. Uranium isotope fractionation. *Rev. Mineral. Geochem* **82**, 799–850 (2017).

9 Stirling, C. H., Andersen, M. B., Potter, E.-K. & Halliday, A. N. Low-temperature isotopic fractionation of uranium. *Earth and Planetary Science Letters* **264**, 208–225 (2007).

10 Andersen, M. *et al.* Closing in on the marine $^{238}U/^{235}U$ budget. *Chem. Geol.* **420**, 11–22 (2016).

11 Tissot, F. L. & Dauphas, N. Uranium isotopic compositions of the crust and ocean: age corrections, U budget and global extent of modern anoxia. *Geochimica et Cosmochimica Acta* **167**, 113–143 (2015).

12 Weyer, S. *et al.* Natural fractionation of $^{238}U/^{235}U$. *Geochimica et Cosmochimica Acta* **72**, 345-359 (2008).

13 Rolison, J. M., C.H.Stirling, Middag, R. & Rijkenberg, M. J. A. Uranium stable isotope fractionation in the Black Sea: Modern calibration of the $^{238}U/^{235}U$ paleoredox proxy. . *Geochimica et Cosmochimica Acta* **203**, 69–88 (2017).

14 Bains, S., Norris, R. D., Corfield, R. M. & Faul, K. L. Termination of global warmth at the Palaeocene/Eocene boundary through productivity feedback. *Nature* **407**, 171-174 (2000).

15 Yamaguchi, T. & Norris, R. D. Deep-sea ostracode turnovers through the Paleocene–Eocene thermal maximum in DSDP Site 401, Bay of Biscay, North Atlantic. *Mar. Micropaleontol.* **86**, 32-44 (2012).

16 Remmelzwaal, S. R. *et al.* Investigating ocean deoxygenation during the PETM through the Cr isotopic signature of foraminifera. *Paleoceanography and Paleoclimatology* **34**, 917-929 (2019).

17 Bornemann, A. *et al.* Persistent environmental change after the Paleocene–Eocene Thermal Maximum in the eastern North Atlantic. *Earth and Planetary Science Letters* **394**, 70-81 (2014).

18 Carmichael, M. J. *et al.* Hydrological and associated biogeochemical consequences of rapid global warming during the Paleocene-Eocene Thermal Maximum. *Global and Planetary Change* **157**, 114-138, doi:https://doi.org/10.1016/j.gloplacha.2017.07.014 (2017).

19 Arreguín-Rodríguez, G. J., Alegret, L. & Thomas, E. Late Paleocene-middle Eocene benthic foraminifera on a Pacific seamount (Allison Guyot, ODP Site 865): Greenhouse climate and superimposed hyperthermal events. *Paleoceanography* **31**, 346-364 (2016).

20 Takeda, K. & Kaiho, K. Faunal turnovers in central Pacific benthic foraminifera during the Paleocene–Eocene thermal maximum. *Palaeogeography, Palaeoclimatology, Palaeoecology* **251**, 175-197 (2007).

21 Clarkson, M. O., Müsing, K., Andersen, M. B. & Vance, D. Examining pelagic carbonate-rich sediments as an archive for authigenic uranium and molybdenum isotopes using reductive cleaning and leaching experiments. *Chem. Geol.* **539**, 119412, doi:https://doi.org/10.1016/j.chemgeo.2019.119412 (2020).

22 Carpentier, M., Weis, D. & Chauvel, C. Large U loss during weathering of upper continental crust: the sedimentary record. *Chem. Geol.* **340**, 91-104 (2013).

23 Lau, K. V., Lyons, T. W. & Maher, K. Uranium reduction and isotopic fractionation in reducing sediments: Insights from reactive transport modeling. *Geochimica et Cosmochimica Acta* (2020).

24 Zhang, F. *et al.* Global-ocean redox variations across the Smithian-Spathian boundary linked to concurrent climatic and biotic changes. *Earth-Sci. Rev.* (2018).

25 Zhang, F. *et al.* Congruent Permian-Triassic δ238U records at Panthalassic and Tethyan sites: Confirmation of global-oceanic anoxia and validation of the U-isotope paleoredox proxy. *Geology* **46**, 327-330 (2018).

26 Clarkson, M. O. *et al.* Uranium isotope evidence for two episodes of deoxygenation during Oceanic Anoxic Event 2. *Proceedings of the National Academy of Sciences* **115**, 2918-2923 (2018).

27 Brennecka, G. A., Herrmann, A. D., Algeo, T. J. & Anbar, A. D. Rapid expansion of oceanic anoxia immediately before the end-Permian mass extinction. *Proceedings of the National Academy of Sciences of the United States of America* **108**, 17631–17634, doi:10.1073/pnas.1106039108 (2011).

28 Zhang, F. *et al.* Multiple episodes of extensive marine anoxia linked to global warming and continental weathering following the latest Permian mass extinction. *Science advances* **4**, e1602921 (2018).

29 Gutjahr, M. *et al.* Very large release of mostly volcanic carbon during the Palaeocene-Eocene Thermal Maximum. *Nature* **548**, 573 (2017).

30 Penman, D. E. & Zachos, J. C. New constraints on massive carbon release and recovery processes during the Paleocene-Eocene Thermal Maximum. *Environmental Research Letters* **13**, 105008 (2018).

31 Bralower, T. *et al.* Late Paleocene to Eocene paleoceanography of the equatorial Pacific Ocean: Stable isotopes recorded at Ocean Drilling Program Site 865. Allison Guyot. *Paleoceanography* **10**, 841-865 (1995).

32 Nunes, F. & Norris, R. D. Abrupt reversal in ocean overturning during the Palaeocene/Eocene warm period. *Nature* **439**, 60-63 (2006).

33 Kennett, J. P. in *Proceedings of the Ocean Drilling Program, Scientific Results.* 937-962 (College Station).

34 Stott, L. D., Sinha, A., Thiry, M., Aubry, M.-P. & Berggren, W. A. Global δ13C changes across the Paleocene-Eocene boundary: criteria for terrestrial-marine correlations. *Geological Society, London, Special Publications* **101**, 381-399 (1996).

35 Thomas, D. J., Zachos, J. C., Bralower, T. J., Thomas, E. & Bohaty, S. Warming the fuel for the fire: Evidence for the thermal dissociation of methane hydrate during the Paleocene-Eocene thermal maximum. *Geology* **30**, 1067-1070 (2002).

36 Kelly, D. C., Nielsen, T. M. & Schellenberg, S. A. Carbonate saturation dynamics during the Paleocene–Eocene thermal maximum: Bathyal constraints from ODP sites 689 and 690 in the Weddell Sea (South Atlantic). *Marine Geology* **303**, 75-86 (2012).

37 Bains, S., Corfield, R. M. & Norris, R. D. Mechanisms of climate warming at the end of the Paleocene. *Science* **285**, 724-727 (1999).

38 Edgar, K. M., Anagnostou, E., Pearson, P. N. & Foster, G. L. Assessing the impact of diagenesis on δ11B, δ13C, δ18O, Sr/Ca and B/Ca values in fossil planktic foraminiferal calcite. *Geochimica et Cosmochimica Acta* **166**, 189-209, doi:https://doi.org/10.1016/j.gca.2015.06.018 (2015).
